# *Brevundimonas vesicularis* (S1T13) Mitigates Drought-Stress-Associated Damage in *Arabidopsis thaliana*

**DOI:** 10.3390/ijms242316590

**Published:** 2023-11-22

**Authors:** Can Thi My Tran, Tiba Nazar Ibrahim Al Azzawi, Murtaza Khan, Sajid Ali, Yong-Sun Moon, Byung-Wook Yun

**Affiliations:** 1 Department of Applied Biosciences, Kyungpook National University, Daegu 41566, Republic of Korearedflower660@yahoo.com (T.N.I.A.A.); 2 Department of Horticulture and Life Science, Yeungnam University, Gyeongsan 38541, Republic of Korea; murtazakhan.bio@gmail.com (M.K.); hangulmys@ynu.ac.kr (Y.-S.M.)

**Keywords:** drought stress, PGPRB, antioxidants, gene expression, ABA, *Arabidopsis*

## Abstract

Drought stress is a significant threat to agricultural productivity and poses challenges to plant survival and growth. Research into microbial plant biostimulants faces difficulties in understanding complicated ecological dynamics, molecular mechanisms, and specificity; to address these knowledge gaps, collaborative efforts and innovative strategies are needed. In the present study, we investigated the potential role of *Brevundimonas vesicularis* (S1T13) as a microbial plant biostimulant to enhance drought tolerance in *Arabidopsis thaliana*. We assessed the impact of S1T13 on Col-0 wild-type (WT) and *atnced3* mutant plants under drought conditions. Our results revealed that the inoculation of S1T13 significantly contributed to plant vigor, with notable improvements observed in both genotypes. To elucidate the underlying mechanisms, we studied the role of ROS and their regulation by antioxidant genes and enzymes in plants inoculated with S1T13. Interestingly, the inoculation of S1T13 enhanced the activities of GSH, SOD, POD, and PPO by 33, 35, 41, and 44% in WT and 24, 22, 26, and 33% in *atnced3*, respectively. In addition, S1T13 upregulated the expression of antioxidant genes. This enhanced antioxidant machinery played a crucial role in neutralizing ROS and protecting plant cells from oxidative damage during drought stress. Furthermore, we investigated the impact of S1T13 on ABA and drought-stress-responsive genes. Similarly, S1T13 modulated the production of ABA and expression of *AO3*, *ABA3*, *DREB1A*, and *DREB2A* by 31, 42, 37, 41, and 42% in WT and 20, 29, 27, 38, and 29% in *atnced3*. The improvement in plant vigor, coupled with the induction of the antioxidant system and modulation of ABA, indicates the pivotal role of S1T13 in enhancing the drought stress tolerance of the plants. Conclusively, the current study provides valuable insights for the application of multitrait S1T13 as a novel strain to improve drought stress tolerance in plants and could be added to the consortium of biofertilizers.

## 1. Introduction

Plants are sessile organisms that face a variety of biotic and abiotic stresses that affect their growth, productivity, and survival [1]. Abiotic stressors include drought, waterlogging, extreme temperatures, salinity, and heavy metals (HMs) [2]. Among these abiotic stressors, drought stress is one of the most common and severe, with several detrimental effects on plant growth and productivity. Drought stress occurs for various reasons, including low rainfall, high temperatures, or soil water deficits [3]. Plants undergo physiological and biochemical changes during drought stress to conserve water and survive under limited water availability. These changes include the closure of stomata to reduce water loss, reduced photosynthesis, and increased production of stress hormones, such as abscisic acid (ABA). Drought stress adversely affects plants, which can vary depending on the severity and duration of the stress, plant species, and cultivars [4]. Therefore, different techniques are needed to enhance drought stress tolerance in different plants. Enhancing induced systemic tolerance (IST) in plants by applying abiotic and biotic elicitors is one of the most significant and interminable phenomena to increase drought stress tolerance [5,6]. After the initiation of IST by any elicitor, plants gain the ability to withstand drought stress throughout their lifespan. The application of elicitors, whether biotic or abiotic, has been globally employed to enhance drought stress tolerance in plants. However, additional research is required to comprehensively grasp the plant defense mechanisms against abiotic stress conditions such as drought stress [7,8].

Reactive oxygen species (ROS), reactive nitrogen species (RNS), and melatonin (MEL) are significant abiotic elicitors that increase the tolerance of plants to biotic and abiotic stressors, including drought stress [9,10]. Sohag, et al. [11] stated that the application of salicylic acid (SA) and hydrogen peroxide (H_2_O_2_) enhanced the drought stress tolerance of rice plants by inducing antioxidant enzyme activities. Chavoushi, et al. [12] suggested that applying SA and sodium nitroprusside (SNP, a nitric oxide donor) enhanced the drought stress tolerance of safflower by reducing free radicle production and lipid peroxidation. Studies have suggested that the application of MEL increased the thermo tolerance of soybeans by activating antioxidants and reducing oxidative stress. Furthermore, the MEL treatment enhanced the thermotolerance of soybeans by altering the production of ABA and ABA-pathway-related genes. Similarly, plant growth-promoting rhizobacteria (PGPR) are also used as a biofertilizer and biocontrol agent to enhance crop productivity and mitigate drought stress [13,14].

PGPR is a group of beneficial bacteria that colonize the rhizosphere (root zone) of plants and promote plant growth and health through various mechanisms [15,16]. PGPR can benefit plants by enhancing their tolerance to abiotic stress, such as drought stress [3,17]. PGPR enhances the defense system of plants against drought stress by inducing antioxidant activities and reducing reactive oxygen species (ROS) production [18]. Furthermore, PGPR increases the expression of abiotic stresses, including drought-stress-related genes and hormones, to enhance the drought stress tolerance of plants [7,16,19,20]. Rathi and Yogalakshmi [21] reported the application of *B. diminuta* MYS6 as an efficient bioinoculant for copper (Cu) remediation and growth promotion of sunflowers. Their study revealed that the inoculation of *B. diminuta* MYS6 enhanced exopolysaccharides production, removed a significant amount of Cu, and improved plant growth, biomass, and chlorophyll under stressful conditions. Similarly, Ganesh, et al. [22] revealed the potential role of PGPR, including *Brevundimonas*, for plant health improvement on model and crop plants.

In the current study, we aimed to investigate the potential role of newly characterized *B. vesicularis* (S1T13) as a microbial plant biostimulant on *A. thaliana* under drought stress. Our objectives paved the way for investigating the role of *B. vesicularis* in enhancing the drought stress tolerance of *A. thaliana* by modulating physiological, biochemical, and molecular parameters such as plant vigor, chlorophyll content (SPAD), chlorophyll *a* and *b* content, ABA production, electrolyte leakage, superoxide anion and H_2_O_2_ production, antioxidant activities and gene expression, and expression of drought-responsive genes.

## 2. Results

### 2.1. B. vesicularis (S1T13) Inoculation Improved Plant Vigor and Chlorophyll (SPAD) Content under Normal and Drought Stress Conditions

**Figure 1:**
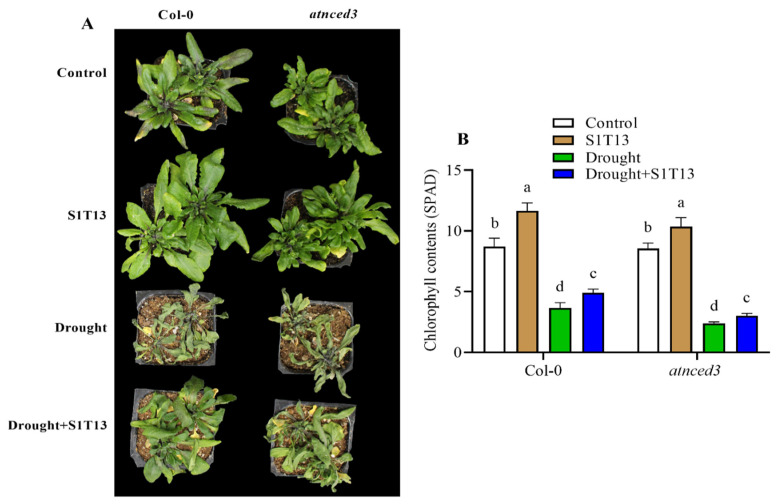
Effects of *B. vesicularis* (S1T13) on Col-0 and *atnced3* plants under normal and drought stress: (**A**) phenotypic evaluation and (**B**) chlorophyll content (SPAD). Letters (a–d) in figure represents the significant difference in each treatment of Col-0 and *atnced3*.

According to the primary screening of Moon and Ali [23], *B. vesicularis* (S1T13) exhibits multitrait plant growth-promoting activities, such as phosphate solubilization and production of phytohormones, siderophores, and 1-aminocyclopropane-1-carboxylic acid (ACC) deaminase. Therefore, this study examined the role of S1T13 in plant vigor and chlorophyll content (SPAD) under normal and drought stress conditions in Col-0 and ABA-deficient mutant line *atnced3*. The amount of chlorophyll in plants is an essential indicator of their photosynthetic capacity and overall health during drought stress. A SPAD (soil plant analysis development) meter is a commonly used device that measures the chlorophyll content in plants, providing valuable information for researchers, farmers, and agronomists. The results showed that under normal conditions, the application of S1T13 improved the vigor and chlorophyll (SPAD) content of Col-0 by 34% and *atnced3* by 21% compared with their controls (treated with water only) (Figure 1A,B). On the other hand, drought stress adversely affected the vigor and chlorophyll (SPAD) content of Col-0 by 58% and *atnced3* plants by 72% compared with their controls (treated with water only) (Figure 1A,B). On the other hand, the inoculation of S1T13 improved plant vigor and enhanced the chlorophyll (SPAD) content of Col-0 and *atnced3* plants by 34% and 25%, respectively, compared with the drought-stressed control plants (Figure 1A,B).

### 2.2. B. vesicularis (S1T13) Inoculation Improved Chlorophyll a and b Content under Normal and Drought Stress Conditions

**Figure 2:**
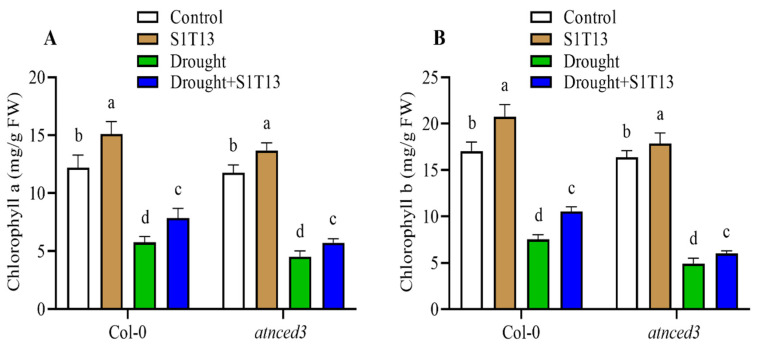
Effect of *B. vesicularis* (S1T13) on Col-0 and *atnced3* plants under normal and drought stress: (**A**) chlorophyll *a* and (**B**) chlorophyll *b* content. Letters (a–d) in figure represents the significant difference in each treatment of Col-0 and *atnced3*.

Chlorophyll *a* and *b* are two primary types of chlorophyll found in plants and are essential for photosynthesis. Therefore, this study evaluated the role of S1T13 in Col-0 and *atnced3* plants under normal and drought stress. The results showed that under normal conditions, S1T13 inoculation increased chlorophyll *a* and *b* content in Col-0 by 24 and 22%, and in *atnced3* plants by 16 and 9%, respectively, compared with their controls (treated with water only) (Figure 2A,B). On the other hand, drought stress significantly reduced chlorophyll *a* and *b* content in Col-0 by 52 and 55% and *atnced3* plants by 61 and 70%, respectively, compared with the controls (treated with water only) (Figure 2A,B). The application of S1T13 significantly reduced the negative effects of drought stress on chlorophyll *a* and *b* content in Col-0 by 37% and 40%, and in *atnced3* plants by 26% and 22%, respectively, compared with the drought-stressed control plants (Figure 2A,B).

### 2.3. B. vesicularis (S1T13) Inoculation Improved the Recovery of Plants after Drought Stress

**Figure 3:**
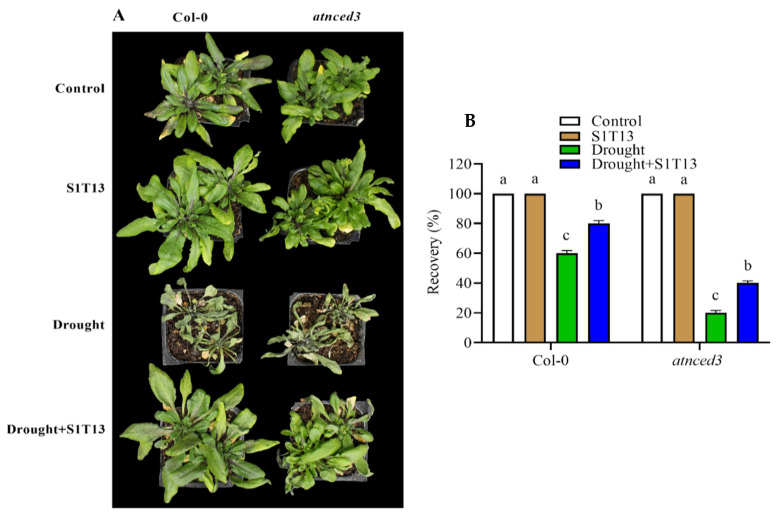
Role of *B. vesicularis* (S1T13) in recovering the Col-0 and *atnced3* plants after drought stress: (**A**) phenotypic evaluation and (**B**) recovery percentage after drought stress. Letters (a–c) in figure represents the significant difference in each treatment of Col-0 and *atnced3*.

The recovery percentage of plants after drought stress can vary considerably depending on various factors: the severity and duration of drought stress, the species and genotype of the plants, and the availability of water, nutrients, and beneficial PPGPR for recovery. Therefore, the current study also investigated the role of S1T13 in the plant recovery percentage after drought stress. These results suggest that after drought stress, the recovery percentage of Col-0 and *atnced3* plants was 60 and 20% compared with their controls (treated with water only) (Figure 3A,B). In contrast, after drought stress, the recovery percentage of Col-0 and *atnced3* plants treated with S1T13 was 80 and 40%, respectively, compared with their controls (treated with water only) (Figure 3A,B). These findings showed that the inoculation of S1T13 significantly improved the recovery percentage of Col-0 and *atnced3* plants after drought stress.

### 2.4. Under Drought Stress, the Inoculation of B. vesicularis (S1T13) Reduced the Production of H_2_O_2_, SOA, and Electrolyte Leakage

**Figure 4:**
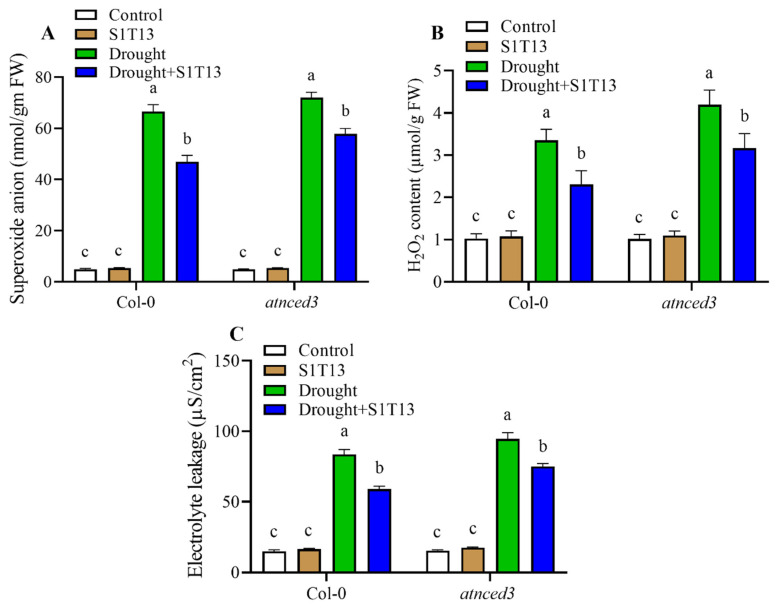
Role of *B. vesicularis* (S1T13) in reducing oxidative stress markers and electrolyte leakage in Col-0 and *atnced3* plants under drought stress: (**A**) superoxide anion, (**B**) hydrogen peroxide (H_2_O_2_), and (**C**) electrolyte leakage. Letters (a–c) in figure represents the significant difference in each treatment of Col-0 and *atnced3*.

During drought stress, plants can experience the accumulation of ROS, such as H_2_O_2_ and SOA, and the accumulation of these ROS causes oxidative damage to plants. Similarly, EL is another important indicator of drought stress and can be used to assess membrane damage in plants. Plants overcome these damages by activating defense systems, such as the antioxidant system. The inoculation of beneficial microbes could enhance the defense system of the plants to reduce H_2_O_2_, SOA, and EL production. Therefore, this study also investigated the role of S1T13 in reducing H_2_O_2_, SOA, and EL. These findings showed that drought stress significantly increased H_2_O_2_, SOA, and EL in Col-0 production by 226, 1265, and 457%, and in *atnced3* plants by 314, 1387, and 510%, respectively, compared with the control plants (treated with water only) (Figure 4A–C). On the other hand, the application of S1T13 reduced the H_2_O_2_, SOA, and EL production in Col-0 by 31, 29, and 29%, and in *atnced3* plants by 24, 20 and 21%, respectively, compared with the drought-stressed control plants (Figure 4A–C).

### 2.5. B. vesicularis (S1T13) Inoculation Enhanced Antioxidant Activities under Drought Stress

**Figure 5:**
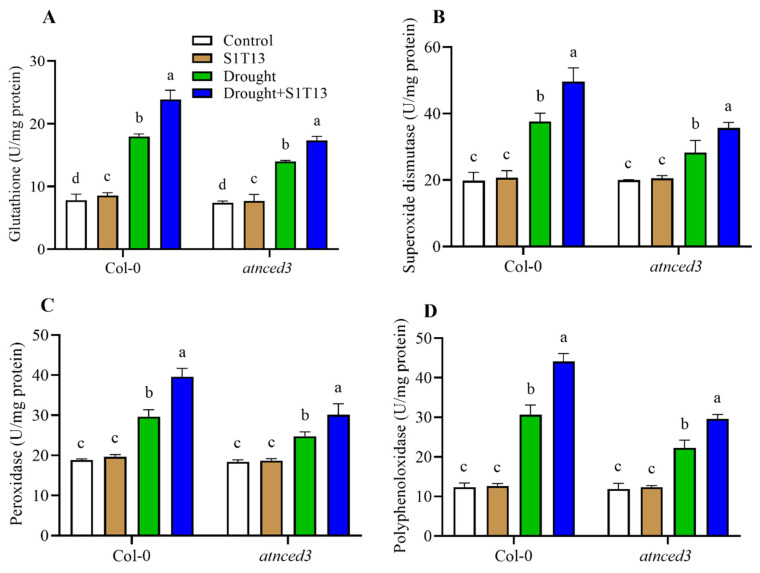
Effect of *B. vesicularis* (S1T13) on the induction of antioxidants in Col-0 and *atnced3* plants under drought stress: (**A**) glutathione, (**B**) superoxide dismutase, (**C**) peroxidase, and (**D**) polyphenoloxidase. Letters (a–d) in figure represents the significant difference in each treatment of Col-0 and *atnced3*.

During drought stress, plants can experience oxidative stress caused by the accumulation of ROS caused by water deficiency and metabolic alterations. On the other hand, plants protect themselves from the adverse effects of drought stress by activating different antioxidants, such as glutathione, SOD, POD, and PPO. In addition, during drought, the antioxidant system could be improved further via the inoculation of beneficial microbes. Therefore, this study examined the role of S1T13 in inducing antioxidant activities during drought stress in Col-0 and *atnced3* plants. These results showed that in response to drought stress, the activities of glutathione, SOD, POD, and PPO were increased in Col-0 by 129, 61, 90, and 148%, and in *atnced3* plants by 89, 35, 41 and 88%, respectively, compared with the control plants (treated with water only) (Figure 5A–D). In response to drought stress, however, the inoculation of S1T13 enhanced the activities of the studied antioxidants in Col-0 by 33, 35, 41, and 44%, and in *atnced3* plants by 24, 22, 26, and 33%, respectively, compared with the drought-stressed control plants (Figure 5A–D).

### 2.6. B. vesicularis (S1T13) Inoculation Increased the Expression of Antioxidant Genes under Drought Stress

**Figure 6:**
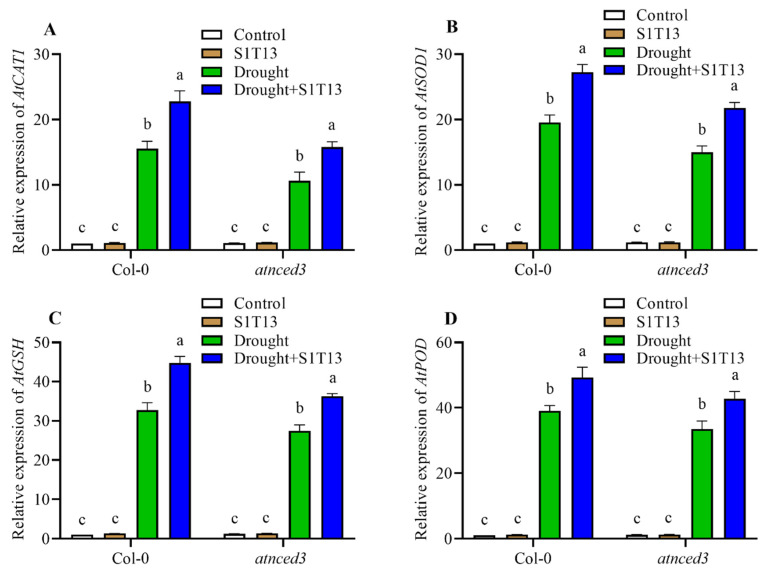
Effect of *B. vesicularis* (S1T13) on the induction of antioxidant genes in Col-0 and *atnced3* plants under drought stress: (**A**) *AtCAT1*, (**B**) *AtSOD1*, (**C**) *AtGSH*, and (**D**) *AtPOD*. Letters (a–c) in figure represents the significant defference in each treatment of Col-0 and *atnced3*.

Our findings revealed that inoculating S1T13 in plants resulted in increased expression of antioxidant genes such as catalase (CAT), SOD, glutathione (GSH), and POD. In Col-0, the inoculation of S1T13 increased the expression of *AtCAT1*, *AtSOD1*, *AtGSH*, and *AtPOD* by 43, 39, 32, and 36% compared with the drought-stressed control plants, while in *atnced3*, the inoculation of S1T13 enhanced the expression of the above genes by 37, 34, 24, and 31% compared with the drought-stressed control plants (Figure 6A–D). These antioxidant genes play a significant role in neutralizing ROS generated during drought stress, protecting the plant cells from oxidative damage. The upregulation of these antioxidant genes indicates that the presence of S1T13 enhanced the plant’s ability to cope with drought stress by bolstering its antioxidant defense mechanisms.

### 2.7. B. vesicularis (S1T13) Inoculation Regulated ABA Level under Drought Stress

**Figure 7:**
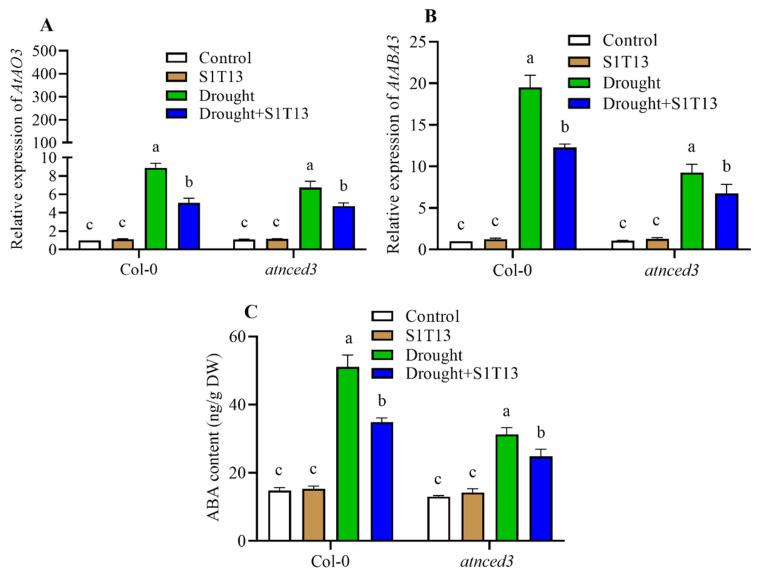
Role of *B. vesicularis* (S1T13) in the modulation of abscisic acid (ABA) in Col-0 and *atnced3* plants under drought stress: (**A**) relative expression of *AtAO3*, (**B**) relative expression of *AtABA3*, and (**C**) ABA contents. Letters (a–c) in figure represents the significant defference in each treatment of Col-0 and *atnced3*.

Abscisic acid (ABA) is pivotal in regulating plant growth and development, particularly in response to environmental stressors, such as drought stress. In addition, PGPR also regulates the production of ABA during drought stress. Therefore, this study also examined the role of S1T13 in regulating ABA during drought stress in Col-0 and *atnced3* mutant plants. These findings showed that drought stress significantly increased *AtAO3* and *AtABA3* expression and ABA production in Col-0 and *atnced3* plants compared with the control plants (treated with water only) (Figure 7A–C). On the other hand, the application of S1T13 modulated the expression of *AtAO3* and *AtABA3* and the production of ABA in Col-0 WT by 43, 37, and 32%, and in *atnced3* plants by 30, 27, and 21%, compared with the drought-stressed control plants (treated with water only) (Figure 7A–C).

### 2.8. B. vesicularis (S1T13) Inoculation Upregulated the Expression of AtDREB1A and AtDREB2A under Drought Stress

**Figure 8:**
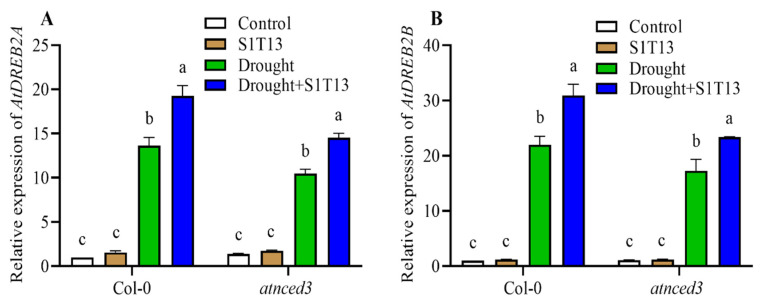
Role of *B. vesicularis* (S1T13) in the induction of *AtDREB* genes during drought stress in Col-0 and *atnced3* plants: (**A**) relative expression of *AtDREB1A* and (**B**) relative expression of *AtDREB2A*. Letters (a–c) in figure represents the significant defference in each treatment of Col-0 and *atnced3*.

The DREB (dehydration-responsive element-binding) family of transcription factors (TFs) plays a crucial role in plant responses to drought stress. These TFs bind to specific DNA sequences known as dehydration-responsive elements and activate the expression of genes involved in stress tolerance. Therefore, the current study examined the role of S1T13 in the induction of *AtDREB1A* and *AtDREB2A* in Col-0 and *atnced3* during drought stress. The results showed that during drought stress, *AtDREB1A* and *AtDREB2A* expression increased in both Col-0 and *atnced3* plants compared with the control plants (treated with water only) (Figure 8A,B). In contrast, the inoculation of S1T13 further increased the expression of both *AtDREB1A* and *AtDREB2A* in Col-0 by 41 and 40%, and in *atnced3* plants by 39 and 35%, respectively, compared with the drought-stressed control plants (Figure 8A,B).

## 3. Discussion

Environmental stresses adversely affect the growth and survival of plants [24,25]. Drought stress is a severe abiotic stress that adversely affects plant growth and yield and even causes the death of plants [26]. Drought stress inhibits plant growth by increasing the production of oxidative stress markers, such as H_2_O_2_, SOA, and EL. Plants induce the activities of antioxidants, such as GSH, SOD, POD, and PPO, to overcome oxidative stress caused by drought stress. In addition, in response to drought stress, plants increase ABA production and the expression of ABA-pathway- and drought-related genes [27,28]. Moreover, the role of rhizobacteria in regulating the growth and ABA concentrations of inoculated plants has been reported with different ABA capacities, as the ability of soil microorganisms to naturally break down phytohormones appears to be a major mechanism in plant–microbe interactions [29]. Overall, these changes reduce plant growth and productivity [30]. Therefore, different techniques are used to enhance the defense system of plants against drought stress, including the application of melatonin, nitric oxide donor, and PGPR [20,28].

This study found that drought stress adversely affected vigor and the chlorophyll content of Col-0 and *atnced3* plants. On the other hand, the application of *B. vesicularis* (S1T13) significantly reduced the adverse effects of drought stress on the vigor and chlorophyll content of Col-0 and *atnced3* plants. Furthermore, under normal conditions, the application of S1T13 also improved the vigor and chlorophyll content of Col-0 and *atnced3* plants (Figure 1 and Figure 2). These results agree with previous work [31], which reported that the application of *Serratia fonticola* and *Lysinibacillus fusiformis* enhanced the salt and drought stress tolerance of cucumber and soybean plants by improving their vigor and chlorophyll content. The current results showed that the application of S1T13 increased the recovery percentage of both Col-0 and *atnced3* plants (Figure 3). These results align with a previous study by Wang, et al. [32], in which they reported that the application of Bacillus cereus significantly enhanced the survival of tomato plants after drought stress.

Drought stress increases superoxide anion and H_2_O_2_ production, which causes oxidative stress and damages the plants. Nevertheless, the application of PGPR reduces the toxic effects of oxidative stress by activating antioxidants such as GSH, SOD, POD, and PPO [28,33]. The current investigation also showed that drought stress increased the production of oxidative stress markers, such as SOA and H_2_O_2_. Applying S1T13 significantly reduced the production of oxidative stress markers by inducing the expression of *AtCAT1*, *AtSOD1*, *AtGSH*, and *AtPOD* genes and the activities of GSH, SOD, POD, and PPO enzymes in both Col-0 and *atnced3* plants (Figure 4, Figure 5 and Figure 6). Similarly, drought stress increases electrolyte leakage in plants. On the other hand, the treatment of PGPR decreases the electrolyte leakage of drought-stressed plants [34]. The present results also align with that study, and the application of S1T13 reduced electrolyte leakage significantly in drought-stressed Col-0 and *atnced3* plants (Figure 4).

Abscisic acid (ABA) plays an essential role in the response of plants to drought stress. PGPR modulates ABA production under abiotic stress, such as salinity and drought [35,36]. The present study showed similar results in that the application of S1T13 regulated the expression of the ABA-pathway-related genes *AtAO3* and *AtABA3* and ABA production in both Col-0 and *atnced3* plants (Figure 7).

The DREB family of TFs is an essential component of the molecular machinery involved in plant drought tolerance [37,38]. By activating the expression of stress-responsive genes and regulating various physiological processes, DREBs help plants adapt to water-deficit conditions and improve their survival and productivity in drought environments [39]. The current study also found that under drought stress, the application of S1T13 significantly increased *AtDREB1A* and *AtDREB2A* accumulation in Col-0 and *atnced3* plants (Figure 8).

Overall, the results showed that an increase in the drought stress tolerance of Col-0 and *atnced3* plants via the application of S1T13 could be due to the role of S1T13 in the improvement of plant vigor, chlorophyll content, antioxidant activities, ABA modulation, *AtDREB1A* and *AtDREB2A* expression, and a reduction in superoxide anion, H_2_O_2_, and electrolyte leakage. Therefore, S1T13 could be used to increase the drought stress tolerance of plants.

Future research into drought tolerance induced by microbial plant biostimulants in model and crop plants will involve conducting field trials at varying drought severity levels, investigating long-term effects, and exploring transcriptomic and proteomic studies. Understanding its interaction with the root microbiome, assessing commercial application feasibility, and exploring genetic manipulation techniques are important aspects. Evaluating its performance under other stress factors in real farming conditions, conducting ecotoxicological studies, and integrating it with crop management practices are also crucial. In a nutshell, this research aims to harness beneficial microbes to improve crop resilience and ensure food security in the face of climate challenges.

## 4. Materials and Methods

### 4.1. Inoculum Preparation

A previous study reported the isolation, evaluation, and identification of multitrait *B. vesicularis* (S1T13) from the coastal sand dune plant species of Pohang Beach, Republic of Korea. *B. vesicularis* (S1T13) has already been reported as having plant growth-promoting traits like production of 1-aminocyclopropane-1-carboxylic acid (ACC) deaminase, indole-3-acetic acid (IAA), siderophore, and phosphate solubilization activities [23]. The partial sequence of the 16S rRNA gene of the strain was submitted to National Center for Biotechnology Information (NCBI) (https://www.ncbi.nlm.nih.gov) with accession number MZ646007 on 28 July 2021, and maintained at 4 °C in an equal volume of nutrient broth and 40% glycerol for future use. In the current experiment, S1T13 was inoculated in Luria–Bertani (LB) broth and incubated at 28 °C for 24 hours (h).

### 4.2. Plant Materials

Seeds of *A. thaliana* Col-0 wild-type (WT) and the *A. thaliana nine-cis-epoxy carotenoid dioxygenase 3* (*AtNCED3*) loss-of-function mutant line *atnced3* were obtained from the Plant Functional Genomics Laboratory at Kyungpook National University, Republic of Korea. Col-0 was used as a WT with a complete set of genomes, while *atnced3* was used as an ABA-deficient mutant line.

### 4.3. Seeds Sterilization, Sowing, Transplantation, PGPR Inoculation, and Samples Collection

Seeds of both genotypes were surface sterilized in 50% commercial bleach with 0.1% Triton X-100 (Sigma Aldrich, Saint Louis, MO, USA) for five minutes (min) as previously described. The seeds were rinsed three times with sterilized distilled water and stratified at 4 °C for 4 h. Sterilized and stratified seeds were grown in seedling trays in a growth chamber under 18/6 h photoperiod at ±22 °C and 60% relative humidity for two weeks. Two-week-old seedlings were transplanted to trays containing autoclaved soil under the same conditions in the growth chamber. After three weeks of transplantation, the bacterial strain was cultured in LB broth, and the OD was measured at 600. The plants other than untreated control (treated with water only) in each well of the tray were inoculated with 10 mL (1 × 10^8^ CFU) of the bacterial suspension. After four weeks, the plants were divided into a control group (only water, only inoculum, and drought-stressed) and an experimental group (drought-stressed + inoculum). The control group (only water and only inoculum) plants were watered regularly for one week. In contrast, the (drought-stressed) and (drought-stressed + inoculum) plants were kept under drought stress by withholding water for seven to 10 days. After drought stress, photographs of the plants were taken for phenotypic evaluation. The survival percentage after drought stress was assessed by rewatering the plants for 24 h, collecting data for survival percentage, and taking photographs for phenotypic evaluation. Furthermore, the samples were collected from the control and experimental plants and stored at −80 °C for further analysis.

### 4.4. Determination of Chlorophyll Content

The chlorophyll content of fully expanded leaves of the control and experimental plants was measured using a soil and plant analysis development meter (SPAD-502, Minolta Co., Ltd., Osaka, Japan). Furthermore, the chlorophyll *a* and *b* contents were measured and calculated. Briefly, 0.5 g of fresh plant material was homogenized in 80% acetone, vortexed for 2 min, and incubated for 30 min at room temperature (RT). The samples were centrifuged for 10 min at 11,000× *g* and 4 °C. The absorbance of the supernatant was measured at 470, 645, and 663 nm using a spectrophotometer (Multiskan GO; Thermo Fisher Scientific, Waltham, MA, USA).

### 4.5. Determination of Superoxide Anion, Hydrogen Peroxide, and Electrolyte Leakage

For superoxide anion (SOA) determination, 0.2 g of fresh leaves were homogenized with 2 mL of 50 mM phosphate buffer at pH 7.8. The homogenized mixture was centrifuged at 10,000× *g* at 4 °C for 15 min. Subsequently, 0.5 mL of supernatant was mixed with 0.1 mL of 10 mM hydroxylamine hydrochloride and 0.5 mL of phosphate buffer (50 mM, pH 7.8) and incubated at RT for 25 min. After incubation, 1 mL of 7 mM naphthylamine and 17 mM sulfanilamide was added to the mixture and further incubated for 30 min at RT. The absorbance was read at 530 nm, and SOA production was calculated with a standard curve of NaNO_2_.

The H_2_O_2_ content was measured using a previously described method [40]. Briefly, 0.2 g of a fresh leaf sample was ground, extracted with 5 mL 0.1% trichloroacetic acid (TAC), and centrifugated at 10,000 rpm for 15 min. The supernatant (0.5 mL) was collected; 1 mL of 1 M potassium iodide and 0.5 mL of 10 mM phosphate buffer (pH 7.0) were added, and the absorbance at 390 nm was measured. The H_2_O_2_ contents (expressed as µmol/g FW) were estimated using the extinction coefficient (ε) = 0.28 mM/cm.

Electrolyte leakage (EL) was determined by collecting 200 mg fresh leaf samples of the control and experimental plants, rinsing carefully with deionized water to remove surface electrolytes, and placing them in test tubes containing 10 mL deionized water for 6 h at RT. After 6 h, electrolyte leakage 1 (EL 1) was measured with a portable conductivity meter (HURIBA Twin Con B-173, Kyoto, Japan). The samples were autoclaved and cooled to RT. Electrolyte leakage (EL 2) was measured using the same conductivity meter. EL was measured using the following formula:

Percent E.L. = EL1/EL2 × 100(1)

### 4.6. Determination of Antioxidant Activities

Glutathione (GSH) was determined using the following methodology. Briefly, 0.2 g fresh leaf samples were ground and homogenized in 3 mL of 5% TAC. After centrifugation, 0.1 mL of supernatant was mixed with 3 mL of 150 mM monosodium phosphate buffer and 0.5 mL Ellman’s reagent and measured using a spectrophotometer at 142 nm.

The superoxide dismutase (SOD), peroxidase (POD), and polyphenol peroxidase (PPO) activities were determined using previously described methods. Briefly, 400 mg fresh leaf samples were crushed using a chilled mortar and pestle. The samples were homogenized with 0.1 M potassium phosphate buffer with 6.8 pH and centrifuged at 5000 rpm at 4 °C for 15 min. Subsequently, the supernatant was a crude enzyme source for determining the activities of SOD, POD, and PPO. The SOD activity was determined as previously described [41], which follows the photo reduction of nitro blue tetrazolium (NBT). The absorbance of the reaction mixture was measured at 540 nm using a spectrophotometer. A unit of SOD is the quantity of enzyme that hampers 50% photo reduction of NBT and is expressed as U/mg of the sample. The POD activity of the reaction mixture containing 50 µL of crude enzyme, 50 µL of pyrogallol (50 µM), 25 µM of H_2_O_2_, and 10 µL of phosphate buffer (0.1 mM, pH 6.8) was determined. The reaction mixture was incubated under dark conditions for 5 min at RT. The reaction mixture was supplied with 25 µL of H_2_SO_4_ (50 *w*/*v*), and absorbance was measured at 420 nm using a spectrophotometer. The PPO activity of a reaction mixture containing 50 µL of crude enzyme extract, 50 µL of pyrogallol (50 µM), and 100 µL of phosphate buffer (0.1 M) was determined. The absorbance at 420 nm was measured using a spectrophotometer, and the activity was calculated using previously described methods [42].

### 4.7. Endogenous Abscisic Acid (ABA) Analysis

The endogenous ABA content was extracted and measured using a previously described method. Briefly, a freeze-dried sample from the control and experimental plants was used to extract the endogenous ABA content using an isopropanol and acetic acid solution with a 95 and 5% ratio, respectively. The standard ABA (20 ng/mL) and filtrate were added to the mixture. Subsequently, all extracts were dried and methylated using diazomethane in preparation for gas chromatography/mass spectrometry–selected ion monitoring (GC/MS–SIM) analysis (6890 N network GC system, and 5973 network mass selective detector from Agilent Technologies, Palo Alto, CA, USA). The responses to the ions of m/e 162 and 190 for Me-ABA and of m/e 166 and 194 for Me-[2H6]-ABA were quantified using a laboratory-based data system software (GraphPad Prism 8.0.1, Thermo Quest, Manchester, UK).

### 4.8. Gene Expression Analysis

Quantitative real-time polymerase chain reaction (qRT-PCR) was used to assess the expression of the genes. Briefly, a TRIzol reagent (Tri-Solution, BSK-BIO, Daegu, Republic of Korea) was used to extract the total RNA from the control and experimental plants according to the manufacturer’s instructions. A BioFACT RT kit (BioFACT, Daejeon, Republic of Korea) was used to synthesize the complementary DNA (cDNA) from approximately 1 µg of RNA. The obtained cDNA was then used as a template for qPCR on an Illumina Eco real-time PCR instrument (Illumina, San Diego, CA, USA) to assess the expression of the selected genes. For each sample, a 2× real-time PCR mix (containing SYBER Green 1 BioFACT, Daejeon, Republic of Korea) was used in a final volume of 20 µL, along with 100 ng of the template DNA and 10 nM of each primer. A no-template control containing nuclease-free water instead of the template DNA was used as a negative control. A 40-cycle PCR was set up with polymerase activation at 95 °C for 15 min, denaturation at 95 °C for 15 s, and annealing and extension at 60 °C for 30 s. The melting curves for each primer pair were evaluated at 60 °C to ensure amplicon specificity, with actin used as the internal reference gene. Supplementary Table S1 lists the primers used in the current study.

### 4.9. Statistical Analysis

Each experiment consisted of 15 plants per treatment, and all treatments were replicated at least three times. The means were derived using data from all replicates from all experiments. Using Statistical Analysis Software (SAS, version 9.1), mean values were compared using Duncan’s multiple range test (DMRT) at a significance threshold of *p* < 0.05. Statistical Analysis System (SAS 9.1) was used for DMRT analysis to evaluate the significance of each treatment. GraphPad Prism (version 6.0, GraphPad, San Diego, CA, USA) was used to visualize the data.

## 5. Conclusions

This study evaluated the beneficial effects of S1T13 in mitigating drought stress in Col-0 and *atnced3* plants. The application of S1T13 had beneficial effects in improving vigor and the chlorophyll content of Col-0 and *atnced3* plants under normal and stressful conditions. Furthermore, the application of S1T13 also enhanced the recovery percentage of drought-stressed plants. In addition, the S1T13 treatment significantly reduced SOA, H_2_O_2_, and EL production by inducing antioxidants in drought-stressed plants. The application of S1T13 increased the drought stress tolerance of both Col-0 and *atnced3* plants by modulating ABA production and increasing the expression of the *AtDREB1A* and *AtDREB2A* genes. The current study suggests that S1T13 could increase drought stress tolerance in plants in drought-stress-prone areas.

## Data Availability

Data are contained within the article and Supplementary Materials.

## Supplementary Materials

The supporting information can be downloaded at: https://www.mdpi.com/article/10.3390/ijms242316590/s1.
