# Supplementary material for: Brevundimonas vesicularis (S1T13) Mitigates Drought-Stress-Associated Damage in Arabidopsis thaliana
Source: Int J Mol Sci. 2023 Nov 22;24(23):16590. doi: 10.3390/ijms242316590 (PMC10705876; doi:10.3390/ijms242316590)
Supplement: Supplementary file 1 [file ijms-24-16590-s001.zip › ijms-2696192-supplementary.pdf]

Supplementary Table S1. List of the primers used in the current study

No	Gene	Forward sequence (5′→3′)	Reverse sequence (5′→3′)
1	***AtDREB1A***	ACGAGTCTTCGGTTCCTCA	ACAAACCCACTTACCGGAGT
2	***AtDREB2A***	GACCTAAATGGCGACGATGT	TCGAGCTGAAACGGAGGTAT
3	***AtAO3***	TGGTAGAGGAGGTCTCGGAA	GGTTCACCCACTGGATGGTA
4	***AtABA3***	CGTCGTCAGTGGAAGGTTTC	AATTTCACCGGTCAGACCCT
5	***AtCAT1***	TCGGGTGCTCCTGTCTGGAA	CTGGCTCCCCTGGCATGAAC
6	***AtSOD1***	GCAGTGAGGGTGTTACGGGG	CTCAGGGGCACCGTGTGTTT
7	***AtGSH***	TCCGCTTTTCTCGGCCCATC	TTCTCTGCTGCAGCCCTTGC
8	***AtPOD***	TTCGCGTACGATTCACCGGG	GGAGCTACGCTGGGTGTGAC
9	***AtActin 2***	AGGTTCTGTTCCAGCCATC	TTAGAAGCATTTCCTGTGAAC
